# Christmas in the Hospitals: England's Greeting

**Published:** 1916-01-01

**Authors:** 


### CHRISTMAS IN THE HOSPITALS.

### England's Greeting.

" England expects "—You know the line,

What British heart does not?

Nor till the sun forgets to shine

Shall Nelson's signal, half divine,

By Britons be forgot.

" England expects "—my distant sons

Across the echoing sea

Have heard the call, have heard the guns,

The hate-shriek of the murdering Huns,

And mustered home to me.

You, lads of mine, home-born or bred
Beneath a distant sun,
Not scatheless in my cause have sped,
But not in vain has England said
She still expects!
Well done!

County of London War Hospital, Epsom. Xmas, 1915.

